# A secondary wave of neutrophil infiltration causes necrosis and ulceration in lesions of experimental American cutaneous leishmaniasis

**DOI:** 10.1371/journal.pone.0179084

**Published:** 2017-06-07

**Authors:** Alex G. Peniche, Diana L. Bonilla, Gloria I. Palma, Peter C. Melby, Bruno L. Travi, E. Yaneth Osorio

**Affiliations:** 1 Centro Internacional de Entrenamiento e Investigaciones Medicas (CIDEIM), Cali, Valle del Cauca, Colombia; 2 Department of Internal Medicine, Division of Infectious Diseases, University of Texas Medical Branch, Galveston, Texas, United States of America; 3 Departamento de Microbiología, Universidad del Valle, Cali, Valle del Cauca, Colombia; 4 Center for Tropical Diseases, University of Texas Medical Branch, Galveston, Texas, United States of America; National Centre For Cell Science, INDIA

## Abstract

We evaluated the importance of neutrophils in the development of chronic lesions caused by *L*. *Viannia spp*. using the hamster as experimental model of American Cutaneous Leishmaniasis (ACL). Neutrophils infiltrated the lesion within the first six hours post-infection. Inhibition of this early infiltration using a polyclonal antibody or cyclophosphamide was associated with transient parasite control but the protective effect vanished when lesions became clinically apparent. At lesion onset (approximately 10 days p.i.), there was an increased proportion of both uninfected and infected macrophages, and subsequently a second wave of neutrophils infiltrated the lesion (after 19 days p.i.) This second neutrophil infiltration was associated with lesion necrosis and ulceration (R^2^ = 0.75) and maximum parasite burden. Intradermal delivery of N-formylmethionyl-leucyl-phenylalanine (fMLP), aimed to increase neutrophil infiltration, resulted in larger lesions with marked necrosis and higher parasite burden than in mock treated groups (p<0.001 each). In contrast, reduced neutrophil infiltration via cyclophosphamide-mediated depletion led to more benign lesions and lower parasite loads compared to controls (p<0.001 each). Neutrophils of the second wave expressed significantly lower GM-CSF, reactive oxygen species and nitric oxide than those of the first wave, suggesting that they had less efficient anti-leishmania activity. However, there was increased inflammatory cytokines and expression of neutrophil proteases (myeloperoxidase, cathepsin G and elastase) in lesions during the second wave of neutrophil infiltration compared with the levels reached during the first wave (6h p.i.). This suggests that augmented neutrophil proteases and inflammatory cytokines during the secondary wave of neutrophils could contribute to skin inflammation, ulceration and necrosis in ACL. The overall results indicate that neutrophils were unable to clear the infection in this model, and that the second wave of neutrophils played an important role in the severity of ACL.

## Introduction

Cutaneous leishmaniasis (CL) is transmitted by the bite of phlebotomine vectors upon blood feeding on the skin of the mammalian host. Local inflammatory signals induced by infection attract neutrophils that infiltrate tissues through the vascular endothelium [1–3]. The neutrophil infiltrate predominant during the first hours of the infection is later reduced by apoptosis and phagocytosis of these dying cells [2, 4, 5]. After resolution of the acute inflammation, between 3–12 days post-infection (p.i.) [6], infected individuals can remain asymptomatic or develop clinically apparent lesions [7]. Neutrophils are frequently observed in both early and chronic cutaneous leishmaniasis (CL) lesions [8–11], but their role in host defense and pathogenesis is uncertain.

Neutrophils have the capacity to kill *Leishmania* species that cause cutaneous or visceral disease. Parasite killing occurs through the production of reactive oxygen species (ROS), degranulation of proteases, and formation of extracellular DNA traps (NETs) [12–15]. However, the parasite has developed evasion mechanisms to survive the microbicidal activation of neutrophils. Amastigotes escape to non-lytic compartments, avoiding acidic tertiary granules [16]. *Leishmania* promastigotes express nucleases that cleave NETs DNA to escape from the NET toxic effects [17]. *Leishmania* also exploits phagocytosis of apoptotic neutrophils to promote macrophage infection [4] and inhibit antigen presentation of dendritic cells [5, 18–20].

Studies in different strains of mice showed that the genetic background and *Leishmania* specie determine whether neutrophils exacerbate or control the infection. Neutrophils exacerbate the lesions of BALB/c mice infected with *L*. *major* but had no effect in C57BL/6 mice [18, 21, 22]. Conversely, neutrophils are protective in BALB/c infected with *L*. *amazonensis* and *L*. *braziliensis* [23, 24], whereas they contribute to severe disease in both strains of mice infected with *L*. *mexicana* [25]. There is no information on the role of neutrophils in chronic ACL caused by parasites of the *Leishmania Viannia* subgenus, which is the species that causes most cutaneous leishmaniasis cases in the New World [26]. Mice typically develop subclinical or self-resolving infections with *L*. *Viannia spp*., whereas susceptible mice Balb/c infected with the Old World *Leishmania* species *L*. *major*, develop progressive overwhelming cutaneous lesions [27]. This, along with the discordant effects of neutrophils in the outcome of human and experimental CL prompted us to evaluate the contribution of these cells in an established hamster model of chronic ACL [28, 29]. We found that neutrophils infiltrate the site of cutaneous infection in two waves, and the second wave was associated with greater lesion necrosis and ulceration. Augmented expression of neutrophil proteases, inflammatory cytokines and decreased oxidative burst capacity and nitric oxide production were implicated in exacerbated cutaneous lesions during the second wave of neutrophil infiltration.

## Materials and methods

### Infection and clinical evaluation of infected hamsters

All experiments were approved by the CIDEIM institutional animal care and use committee and were performed according to the Guiding Principles for Biomedical Research Involving Animals (Council for International Organizations of Medical Sciences) and the Colombian regulations (Law 84 of 1989, resolution #0084300 of 1993). *Leishmania Viannia panamensis* (MHOM/CO/94/1989) promastigotes were cultured for 7 days in Seneckjie’s medium and were injected intradermally in the snout of male Golden hamsters (*Mesocricetus auratus*) (10^6^ parasites per 50 μL PBS). Lesion size was measured with a digital caliper and expressed as an index (area of the snout / area of the snout before infection) [28, 29]. Ulcer was defined as disrupted skin at the site of infection. Necrosis was identified as accumulation of dry exudate around the ulcer. Clinical severity of the ulcer and necrosis was scored in a qualitative scale as benign (1), moderate (2), or severe (3) [30].

### Histopathological evaluation

Skin lesions were fixed in 10% neutral buffered formalin and embedded in paraffin. Five μm thick sections were stained with hematoxylin-eosin (H&E) and blind histological scores were assigned via microscopy. Cells were counted in 21 quadrants in a standard area of 0.21 mm^2^ at 100X magnification [30, 31]. The frequency of extracellular parasites in the connective tissue was recorded as: absent (0), one or more parasites per standard section (1), ten or more parasites per standard section (2), ≥100 parasites per standard section (3), and ≥1000 parasites per standard section (4). The ulcer and necrosis was scored according to its extension as follows: absent (0), less than ¼ field (1), less than ½ field (2), or more than ½ field [30].

### Establishment of methods to evaluate neutrophil recruitment

Different methods to deplete or promote neutrophil recruitment were evaluated using an air-pouch model as described [32, 33]. A rabbit anti-hamster neutrophil polyclonal antibody was generated as described [34] and its neutrophil-depleting capacity compared to an anti-mouse Gr-1 monoclonal antibody (clone RB6-8C5). Rabbit anti-hamster neutrophil anti-serum (50 μL) was injected in the air pouch 6 or 24 h before injection of 10^6^ *L*. *panamensis* promastigotes and the number of neutrophils and macrophages quantified at various time points up to 72 hrs post-infection. Aminophylline (50–800 mg/kg), Metamizole (500–1000 mg/kg), and Cyclophosphamide (200–400 mg/kg; Baxter), which have been used previously to induce neutropenia [35–40] were also evaluated in the air pouch model. Conversely, induction of neutrophil recruitment was evaluated by instillation of 250–500 μg Lipopolysaccharide (LPS, Sigma), 25 μg N-formyl-methyl-leucine-phenylalanine (fMLP, Sigma), and 0.05–0.1 μg Di-Nitro-chloro-benzene (DNCB, Sigma) 6 hrs before injection of 1x10^6^ parasites/1 mL of PBS in the air pouch.

### Inhibition or stimulation of neutrophil recruitment to CL lesions

To decrease the first wave of neutrophil recruitment either 50 μL of polyclonal anti-serum (ID) or 200 mg/kg of cyclophosphamide (oral) was given 6 h before the ID infection of hamsters. To promote neutrophil recruitment, 25 μg fMLP was injected intradermally (ID) at infection site 6h before parasite inoculation. To aim the second wave of neutrophils the same doses were given between 16 and 22 p.i. every 48h.

### Oxidative function and parasite killing by neutrophils

Neutrophils were isolated from the infected skin at 6 hours p.i. (first wave) or from established lesions (second neutrophil wave) using a Medimachine (BD). To measure hydrogen peroxide (H_2_O_2_) production, cells were incubated 30 min in the dark with 20 μM 2',7'-dichlorofluorescin-diacetate (DCFH-DA, Molecular Probes). The production of superoxide anion (O_2_-) and nitric oxide (NO) were estimated using 5 μM Dihydroethidium (DHE, Sigma) and 0.5 μM 4-amino-5-methylamino-2´7´-difluorofluorescein diacetate (DAF-FM, Molecular probes), respectively. After 30 min of incubation, the percentage of fluorescent positive neutrophils (identified by FSC/SSC) was determined by flow cytometry.

Killing of *Leishmania* was determined in neutrophils isolated from the peritoneal cavity of hamsters. Cells were centrifuged with Dextran T500 followed by density gradient separation using Histopaque^®^-1077. Neutrophils were infected *in vitro* with CSFE-labeled *L*. *panamensis* promastigotes (10μM), incubated for 20 min at 34°C, 5% CO_2_ and then washed by low-speed centrifugation three times with cold PBS. The percentage of apoptotic neutrophils and dead neutrophils was estimated by detection of phosphatidylserine (Annexin V), and propidium iodide (BD) staining of neutrophils similarly infected with unlabeled parasites.

### Protease and cytokine expression

Protease and cytokine expression was measured in uninfected and infected skin at the first and second wave of neutrophil infiltration (6 h and 28 days post-infection, respectively). Primers were designed using the hamster mRNA sequence or predicted RNA sequence of the corresponding gene (NCBI data base, National Center for Biotechnology). Primers were identified using primer-Blast (NCBI) with parameters set to give PCR products >100bp and <250bp, single Tm (>80ᵒC) and spanning different exons (Splign, NCBI) (S1 Table). These proteases are mainly produced by neutrophils with exception of MMP9. RNA was isolated from the skin by treating the tissue with proteinase K (80 μg) for 30 min at 55°C. After protein digestion, RNA was extracted (RNAeasy, Qiagen), and treated with DNase I (Turbo DNase, Thermo Fisher Sci.). After reverse transcription (High Capacity Reverse Transcription Kit, Thermo Fisher Sci.), cDNA was amplified with specific primers and SYBR green (iTaq Universal SYBR green, Bio-Rad) and detected by qPCR (Viia 7 Real time PCR detection system, Applied Biosystems). The fold change of expression of the target gene was calculated by the delta delta CT method using uninfected skin as calibrator and the 18S gene as reference gene.

### Statistical analysis

Data were analyzed using SPSS software (Statistical Software for Social Sciences V. 7.5), or GraphPad Instat3. Tests were chosen according data distribution following software recommendations. Statistical tests and number of animals are described in figure legends. Correlations between variables (Spearman coefficient) were established using GraphPad (Prism5).

## Results

### Neutrophils infiltrate ACL lesions after the infection and after the lesion onset

**Fig 1:**
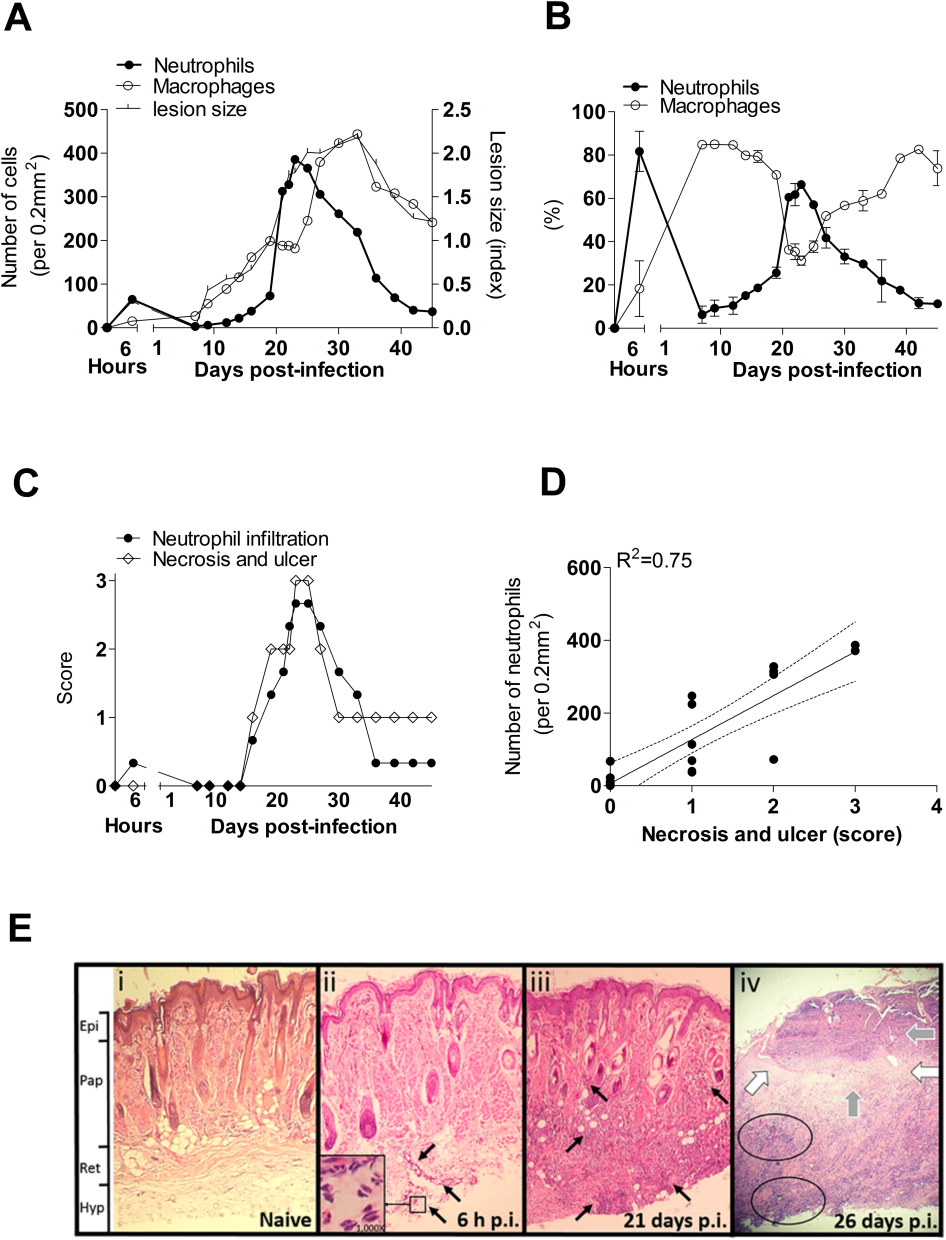
Neutrophils are associated with severity of cutaneous lesions in hamsters infected with *L*. *V*. *panamensis*. (A), Number of neutrophils or macrophages infiltrating the lesion (primary Y-axis) and lesion size (secondary Y-axis) post-infection; (B), Neutrophils during the development of cutaneous lesions (first wave, 2–6 hours after infection; second wave, after 19 days of infection); (C), Neutrophil infiltration and clinical severity of ulcer and necrosis from 0 to 45 days post-infection; (D), Intensity of neutrophil infiltrate and severity of ulcer and necrosis; Determined in a standard area of 0.21mm^2^ in two different samples per time point; (E), Representative histopathology of tissue sections (100X) stained with H&E obtained from snout of hamsters: (i) Naïve tissue without inflammatory infiltrate, (ii) Skin tissue at 6 h p.i. showing presence of neutrophils in reticular dermis (ret) and hypodermis (hyp) (black arrows) and typical tri-lobulated nuclei of neutrophils (inset, 1000X magnification), (iii) Cutaneous lesions at 21 days p.i. showing dense neutrophil infiltrate involving the hypodermis and papillary dermis (pap) (black arrows), (iv) Cutaneous ulcer at 26 days p.i., showing disruption of epidermis (white arrows), disseminated necrosis across the dermis and hypodermis (black circles), with abundant exudative material (grey arrows).

To evaluate the role of neutrophils in the evolution of cutaneous lesions of *L*. *panamensis*, we determined the proportion and number of neutrophils and macrophages infiltrating the lesion at different times after intradermal infection of hamsters. We found that neutrophils infiltrated the lesions in two waves after infection. An early wave of neutrophils occurred between 2–6 hrs post-infection (p.i.) (Fig 1A and 1B). The magnitude of the neutrophil infiltrate during the first peak was modest compared to the massive second peak of neutrophil infiltration after 19 days p.i. (Fig 1A and 1B). The number of neutrophils in the second wave was approximately three-fold greater than in the first wave (Fig 1A), and the timing of the second wave was coincident with the onset and peak of skin necrosis and ulceration (Fig 1C). The number of neutrophils in the second wave correlated with the severity of ulcers and necrosis in the lesions (R^2^ = 0.75, p<0.001, Fig 1D and 1E), and weakly correlated with the lesion size (R^2^ = 0.62, S1A Fig). The maximum lesion size and parasite burden was observed after the peak of the second neutrophil wave began to decrease (30 days p.i.) (Fig 1A and 1B). After this time macrophages became the predominant cells in the chronic lesion until the end of the study period at 46 days p.i. (Fig 1A and 1B). Macrophages, in contrast to neutrophils, strongly correlated with the size of cutaneous lesions (R^2^ = 0.79, S1B Fig) but not with ulceration or necrosis (R^2^ = 0.20).

**Fig 2:**
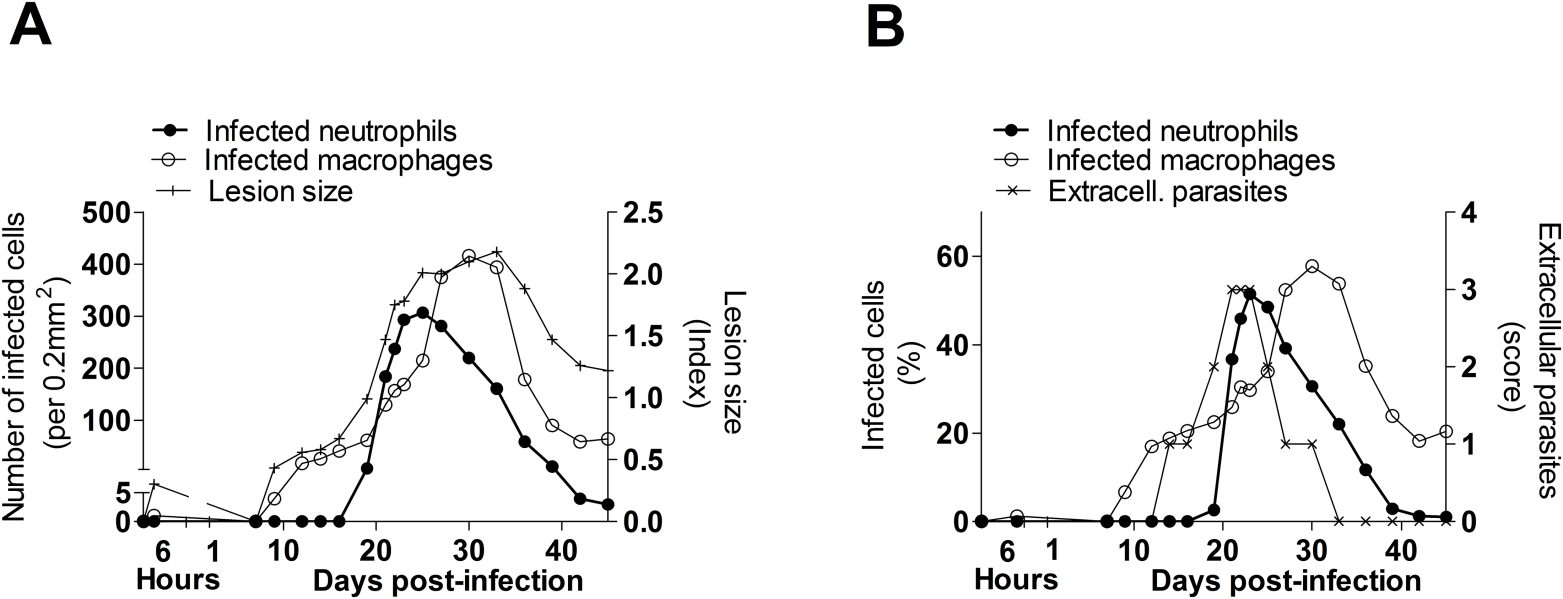
Infected neutrophils and parasite burden in cutaneous lesions of hamsters infected with *L*. *V*. *panamensis*. (A), Number of infected neutrophils or infected macrophages (primary Y-axis) and size of cutaneous lesions (secondary Y-axis) at different times p.i.; (B), Proportion of infected neutrophils, infected macrophages (primary Y-axis), and frequency of extracellular parasites (secondary Y-axis) (Score: 0, No extracellular parasites; 1, one to ten extracellular parasites; 2, ten to one hundred extracellular parasites; 3, one hundred or more extracellular parasites). Tissue sections from cutaneous lesions were stained with H&E and evaluated microscopically in a standard area of 0.21mm^2^ at 100X magnification, n = 2 samples per time point.

To determine the kinetics of neutrophil and macrophage infection, and to determine whether infected cells correlated with the severity of cutaneous lesions, we evaluated the number and proportion of infected cells in skin biopsies over the course of infection. We found that during the first 2–6 hours of infection, dermal neutrophils and macrophages were infrequently infected (0.06% and 0.31% respectively). Neutrophils contained 1 or 2 amastigotes with no signs of division by binary fission at the microscope. At the time of lesion onset (about day 10 p.i.), there was an increase in the number and proportion of infected macrophages, but the number and percent of infected neutrophils did not increase until about 20 days p.i. (Fig 2A and 2B). Extracellular parasites were observed in the interstitial space at this time but became less frequent by 30 days p.i. (Fig 2B). At 30 days p.i. lesions reach maximum size and intracellular parasite burdens (Fig 2A and 2B).

### First wave of neutrophils has no effect in lesion outcome

To confirm the observed association of neutrophils with the severity of necrosis and skin ulceration, we carried out in vivo experiments to increase or reduce neutrophil infiltration. We first conducted a series of experiments using an air pouch model to identify interventions that effectively modulated neutrophil numbers in hamsters [32],[33]. The rabbit anti-hamster neutrophil polyclonal antibody reduced infiltrating neutrophils without affecting the macrophage population (S2A Fig). Treatment of hamsters with cyclophosphamide effectively eliminated neutrophils, but not macrophages, for up to 48 hrs post-treatment (S2B, S2C and S2E Fig). The anti-mouse Gr-1 monoclonal antibody (clone RB6-8C5) depleted both neutrophils and macrophages (S2A Fig). The peptide fMLP enhanced neutrophil recruitment to the air pouch without affecting the macrophage population (S2D and S2E Fig).

**Fig 3:**
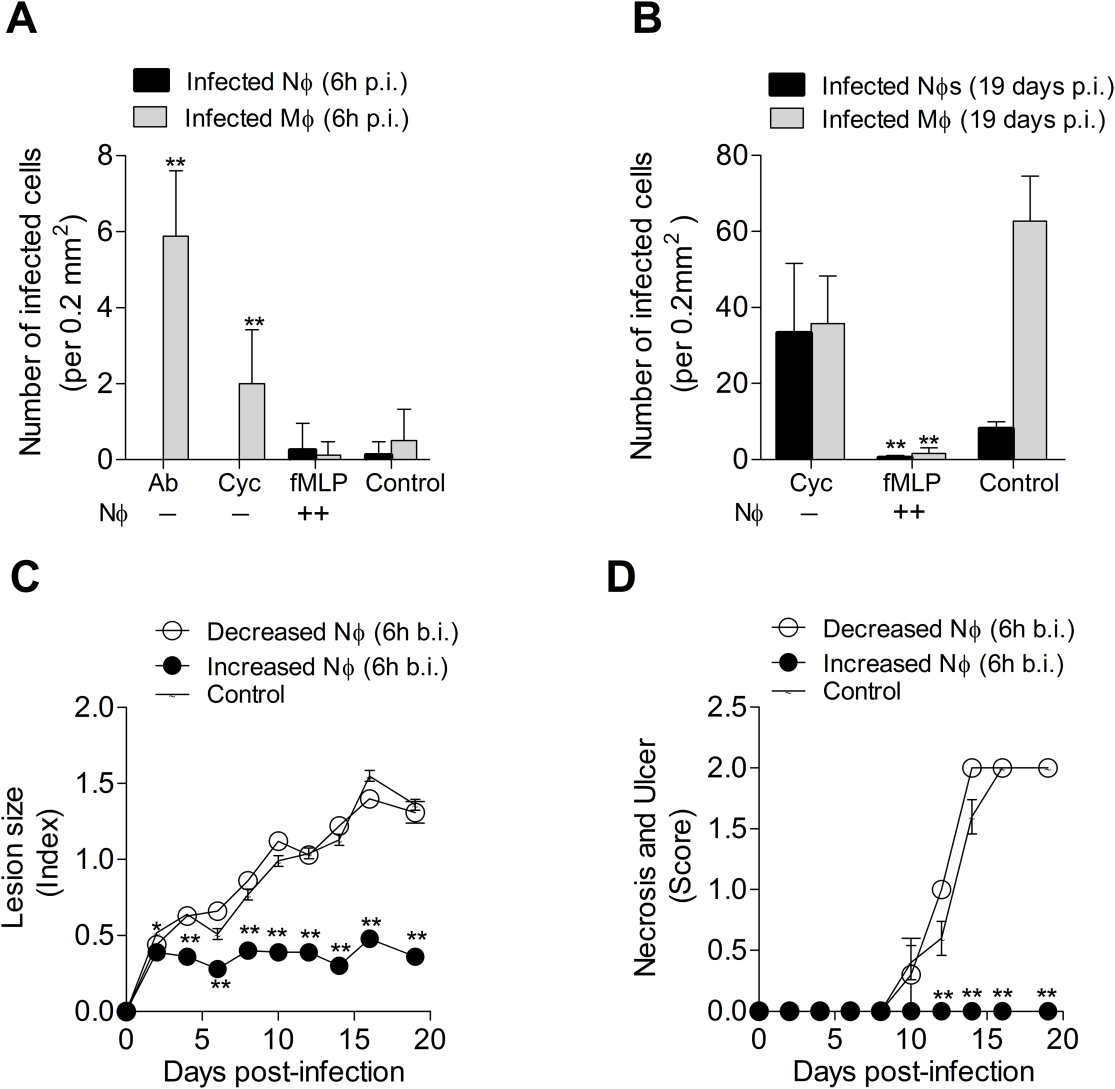
Impact of early neutrophil infiltration in parasite killing and lesion development in hamsters infected with *L*. *V*. *panamensis*. (A), Parasite burden in the cutaneous lesion of hamsters treated to inhibit neutrophil recruitment with either a polyclonal anti-serum (Ab) (6h before infection) or 200mg/kg cyclophosphamide (cycloph.) (24h before infection) or treated to increase neutrophil recruitment with 25 μg fMLP (6h before infection), evaluated at 6h post-infection; (B), Parasite burden in lesion of hamsters treated to inhibit neutrophil with cyclophosphamide or treated to recruit neutrophils with fMLP, evaluated at 19 days p.i. Determined in skin sections stained with H&E at 100X magnification. Number of cells = proportion of cells x total number of cells / 100 (determined in a standard area of 0.21mm^2^ at 100X magnification). (C-D), Lesion size and severity of the skin ulcer and necrosis followed during 19 days of infection. *p<0.05; **p<0.01, with reference to control group, n = 8 hamsters per group.

To determine the effect of neutrophil modulation on clinical lesions caused by *L*. *panamensis*, we treated the animals either with the neutrophil-depleting polyclonal antibody (intradermal) or cyclophosphamide (oral) before infection with *L*. *panamensis*. Prevention of early lesional neutrophil infiltration by either method at the time of *L*. *panamensis* infection resulted in no detectable infected neutrophils, but in a significant increase in the number of infected macrophages at the infection site compared with mock treated hamsters (Fig 3A). However, groups treated to promote neutrophil infiltration with fMLP had parasite burdens equivalent to those of control animals at 6h post-infection (Fig 3A). We performed *in vitro* infections and assessed the capacity of neutrophils to kill *Leishmania* during the first 24h of infection. We found no evidence of parasite killing in neutrophils infected *in vitro* with *L*. *panamensis* during the first 24 hours of infection (S3A Fig). Although we found modest neutrophil apoptosis and cell death at 24h post-infection (S3B and S3C Fig).

To clarify whether the killing of *Leishmania* occurs later in the course of infection, we evaluated the effect of early neutrophil modulation on lesion development at 19 days p.i. (before the second wave of neutrophils). The group subjected to neutrophil depletion at the time of infection developed lesions and harbored parasite burdens equivalent to the control group (Fig 3B, 3C and 3D). Indicating that the initial augmented parasite burden observed at 6h p.i. was transient and did not influence the lesion onset. In contrast, groups that received a single dose of fMLP to increase the influx of neutrophils exhibited more controlled lesions and parasite burdens at 19 days p.i. (Fig 3B, 3C and 3D). This suggests that these fMLP-activated neutrophils controlled the parasites after 6h of infection.

### Second wave of neutrophils exacerbate ACL lesions

**Fig 4:**
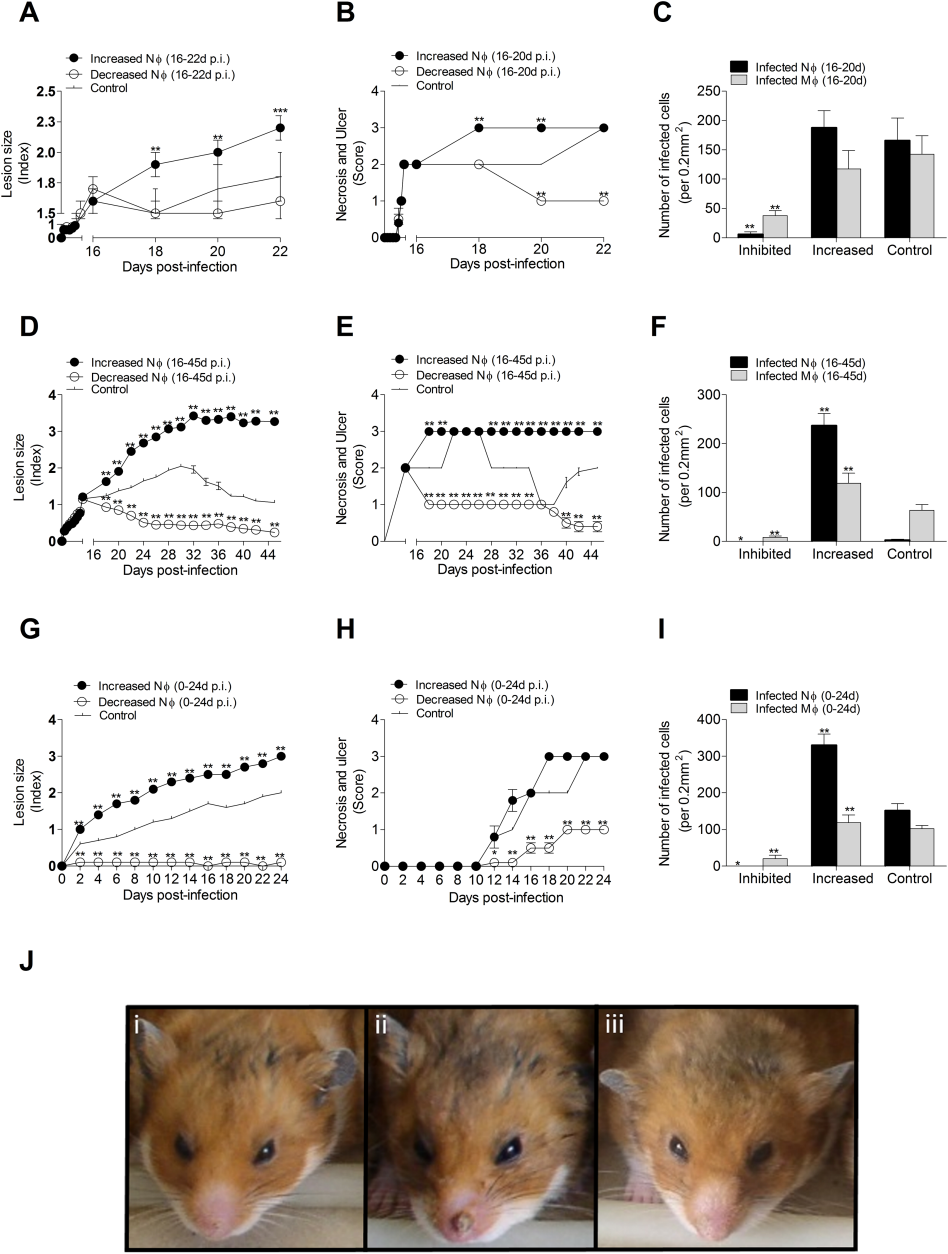
Impact of late neutrophil infiltration on lesion severity and parasite burden in hamsters infected with *L*. *V*. *panamensis*. (A-C), Modulation of the second wave of neutrophils from 16–22 days of infection: showing lesion size (A), severity of skin necrosis and ulcer (B), and parasite burden at 22d p.i. (C) in lesions of hamsters treated to recruit neutrophils with fMLP or treated to inhibit neutrophils with cyclophosphamide; D-F, Modulation of the second wave of neutrophils from 16–45 days of infection: showing lesion size (D), severity of skin necrosis and ulceration (E), and parasite burden at 45 days of infection (F); G-I, Modulation of first and second wave of neutrophil infiltration up to 24 days post-infection showing lesion size (G), severity of skin necrosis and ulcer (H), and parasite burden at 24 days of infection (I). Parasite burden was evaluated in skin sections stained with H&E at 100X magnification. Number of cells = proportion of cells x total number of cells / 100 (determined in a standard area of 0.21mm^2^ at 100X magnification). * p<0.05, **p<0.001, comparison against control group, n = 8 hamsters per group; (J), Representative snout lesions at 22 days p.i.: (i) Control group (PBS), (ii) Increased neutrophil recruitment (fMLP), (iii) Decreased neutrophil recruitment (Cyclophosphamide).

To evaluate the influence of the second wave of neutrophil influx in lesion evolution and parasite load, we compared groups in which neutrophils were depleted with cyclophosphamide or their recruitment/activation was augmented with fMLP between days 16 and 22 p.i. In contrast to the results of modulation of neutrophil numbers early in the course of infection, a fMLP-mediated increase in neutrophils during the second wave of infiltration resulted in larger, more ulcerated, necrotic lesions (Fig 4A and 4B). However, the parasite burden was similar to the control group (Fig 4C). Although animals with fewer neutrophils developed lesions of similar size to controls (Fig 4A), the lesions had reduced ulceration and necrosis (Fig 4B) and a lower parasite burden (Fig 4C). Next, we compared groups in which the second wave of neutrophil infiltration was modulated for a longer period (days16-45 p.i). In line with the previous result, hamsters with fMLP-induced increased neutrophil infiltration had more necrotic and ulcerated lesions and higher parasite burden than controls (Fig 4D–4F). Neutrophil depletion resulted in more benign lesions (Fig 4D and 4E) and diminished parasite burden compared to controls (Fig 4F). Finally, interventions to modulate both the first and second wave of neutrophil infiltration (0–24 days p.i.) gave comparable results to modulation of only the second wave. Depletion of neutrophils in both waves reduced lesion size, skin ulceration and necrosis, and parasite load (Fig 4G–4I). Enhancement of neutrophil infiltration in both waves promoted lesion size and parasite burden, and transiently increased ulceration and necrosis (Fig 4G–4I). Representative pictures of the clinical lesion site are shown in Fig 4J. Collectively, these data indicate that the second wave neutrophil infiltration was the primary driver of ulceration, necrosis, and parasite load.

### Second wave neutrophils have weak oxidative burst capacity

**Fig 5:**
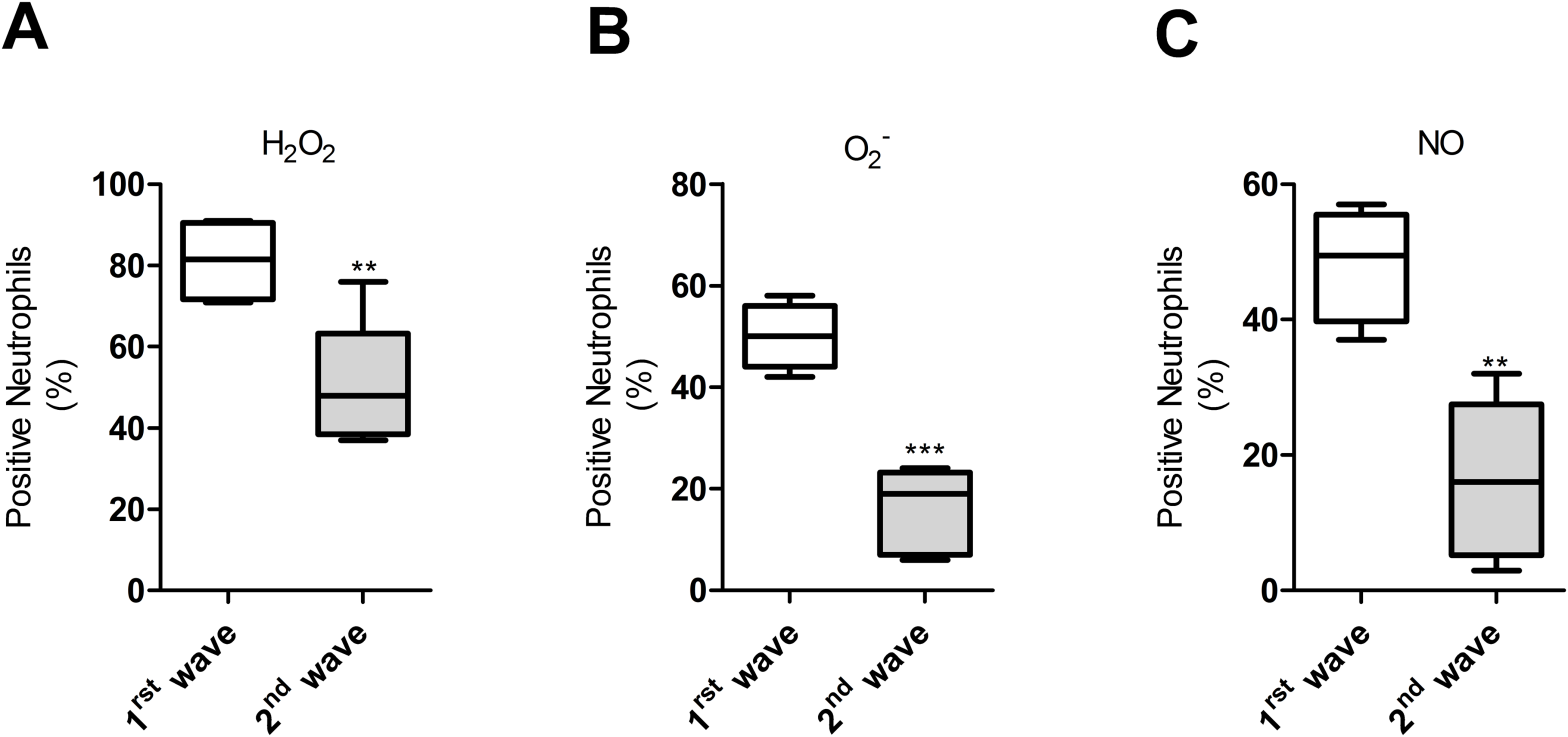
Oxidative burst is decreased in neutrophils during the second wave of lesion infiltration. (A), Frequency of neutrophils expressing hydrogen peroxide (H_2_O_2_), determined with 2',7'-dichlorofluorescin-diacetate (DCFH-DA), **p = 0.007, T-Test); (B), Frequency of neutrophils expressing superoxide anion (O_2_-) determined with Dihydroethidium (DHE, ***p = 0.001,T-Test); (C), Frequency of neutrophils expressing Nitric oxide (NO) determined with 4-amino-5-methylamino-2´7´-difluorofluorescein diacetate (DAF-FM,**p = 0.001, T-Test). Neutrophils from the lesions of hamsters infected with *L*. *V*. *panamensis* were identified in flow cytometry by size and granularity. First wave (7–14 days post-infection), second wave (after 30 days post-infection). Data pooled from 3 different experiments with 2–3 observations per experiment.

The contrasting results observed during the early and late waves of neutrophil infiltration regarding lesion severity and parasite burden prompted us to evaluate neutrophil activation at these time points. We found that the production of hydrogen peroxide (Fig 5A), superoxide anion (Fig 5B), and nitric oxide (Fig 5C) was significantly higher in neutrophils infiltrating lesions during the first hours of the infection (first wave) compared to those of the second wave. This indicated that the anti-leishmanial oxidative capacity of neutrophils present during the second wave was weaker than those of the initial wave.

**Fig 6:**
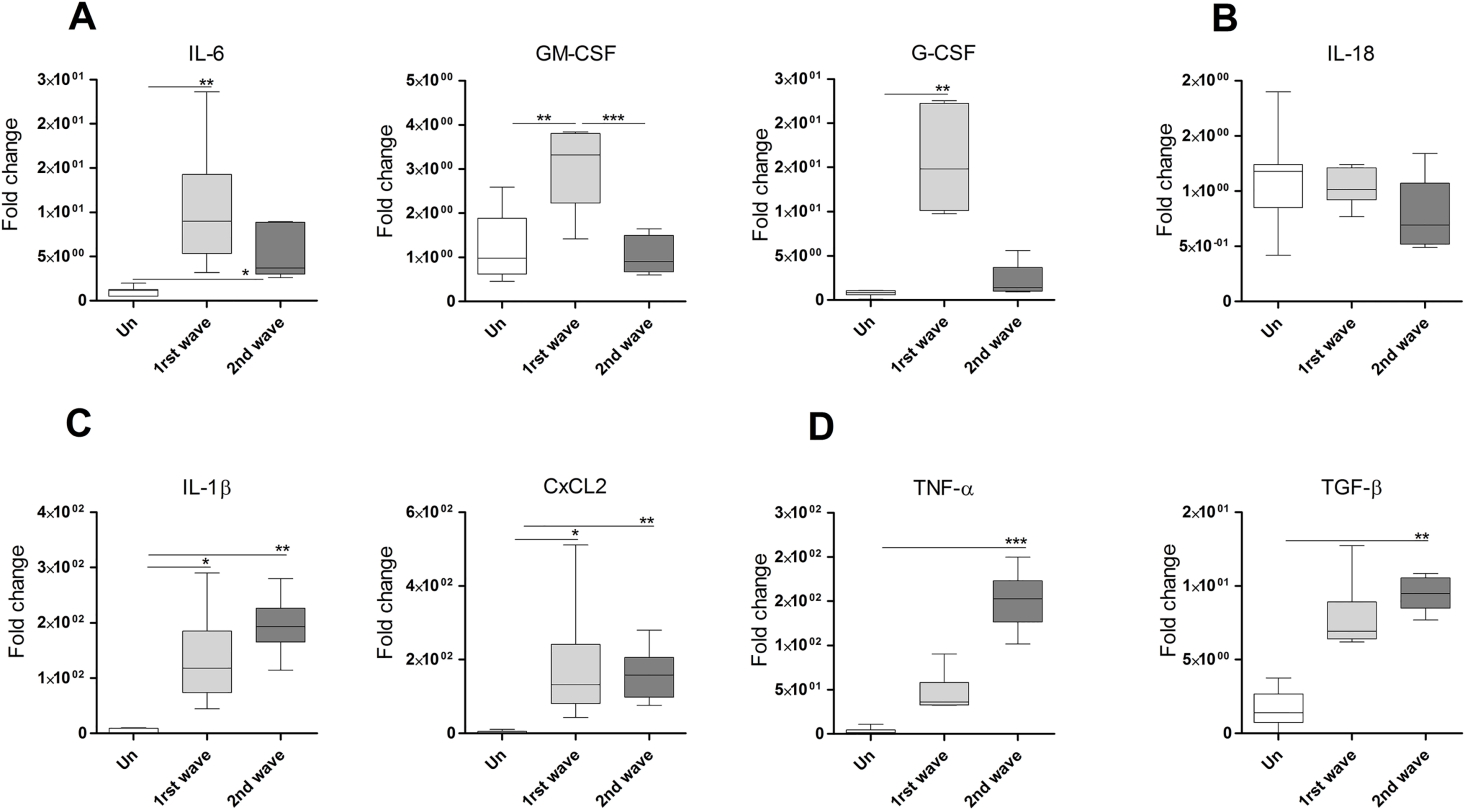
Cytokine and chemokine profile in the cutaneous lesion of hamsters infected with *L*. *panamensis*. (A-D), Expression levels of mRNA in cutaneous lesions during the first wave (6h) or second wave (28d) of neutrophils, determined by qPCR.(*p<0.05, **p<0.01, ***p<0.001, Dunn's Multiple Comparisons Test). Fold change expressed with reference to skin of uninfected animals.

To understand the environment that regulates the oxidative burst and nitric oxide production in the lesion we determined the expression of inflammatory cytokines involved in this regulation. During the first wave of neutrophil infiltration we found augmented IL-6, GM-CSF and G-CSF, which was greater than the expression during the second wave (Fig 6A). Expression levels of IL-18 remain similar in uninfected and infected lesions (Fig 6B) whereas IL-1β and CxCL2 was raised in both waves (Fig 6C). In contrast, TNF-α and TGF-β were increased only during the second wave (Fig 6D).

### ACL lesions over express neutrophil proteases

**Fig 7:**
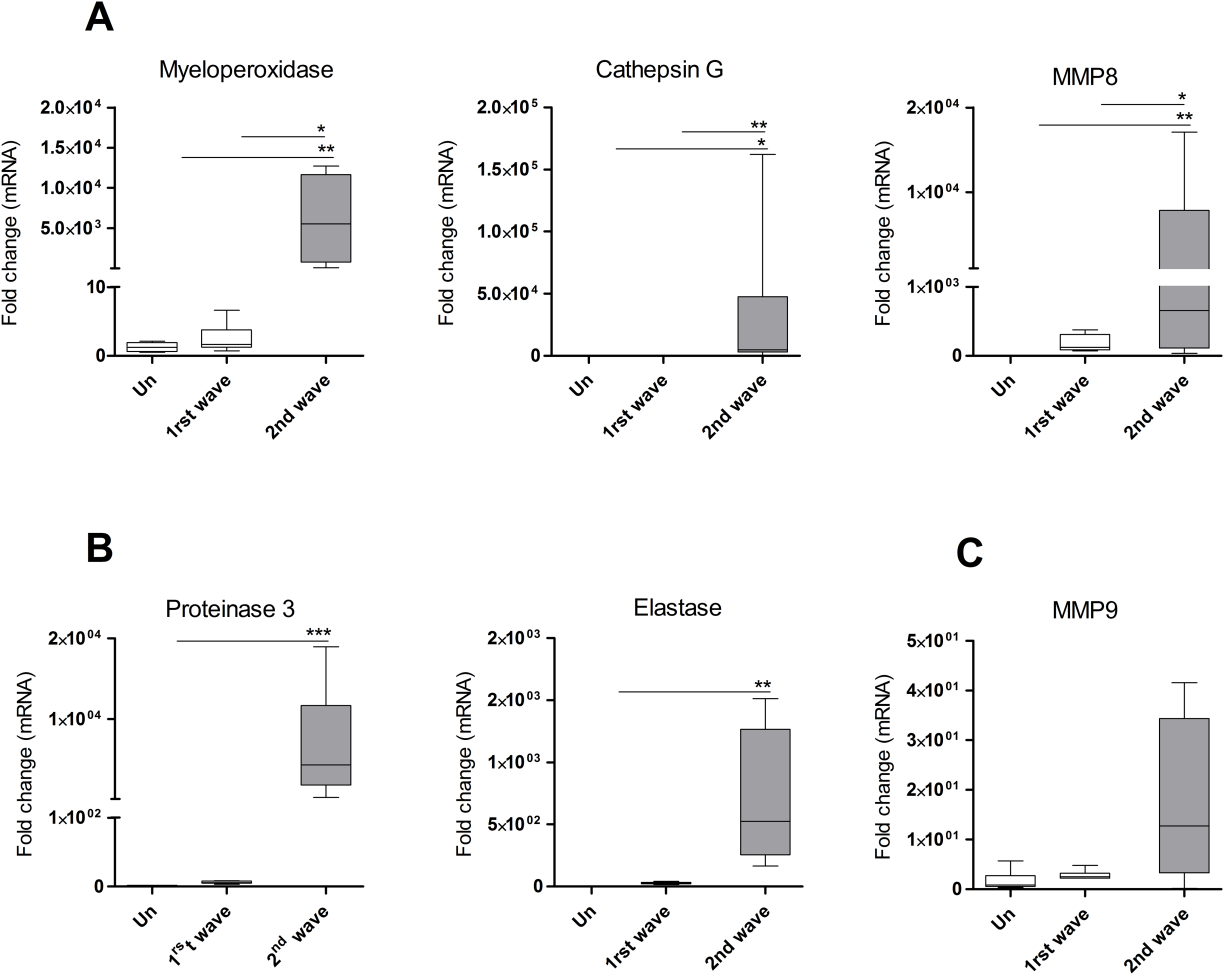
Protease expression was enhanced with the infection and during the second wave of neutrophil infiltration. (A-C), Expression of myeloperoxidase, cathepsin G, matrix metallopeptidase 8 (MMP8), proteinase-3, neutrophil elastase and matrix metallopeptidase 9 (MMP9) determined in skin from uninfected hamsters (Un), or in the infection site of hamsters infected with *L*. *V*. *panamensis*. Lesions obtained during the first wave of neutrophil infiltration (1^st^ wave, 6h p.i.) or during the second wave of neutrophil infiltration (2^nd^ wave, 28d p.i.). Determined by qPCR.*p<0.01; **p<0.001; ***p<0.001, n = 5–7 lesions per group. One-way Anova, Kruskal-Wallis statistic.

Since the increased tissue pathology was not caused by increased generation of ROS and NO, we hypothesized that neutrophil proteases could be interacting with inflammatory cytokines to promote the severity of cutaneous lesions. We developed qPCR assays for hamster proteases and evaluated the expression of proteinase 3, myeloperoxidase (MPO), cathepsin G, matrix metallopeptidase 8 (MMP8), matrix metallopeptidase 9 (MMP9), and neutrophil elastase. We found that expression of all the proteases was increased in chronic lesions compared to skin of uninfected hamsters (Fig 7A and 7B), with the exception of MMP9 (Fig 7C). Expression of MPO, cathepsin G and MMP8 was greater in the second wave of neutrophil infiltration compared to that of the first wave (Fig 7A). Because during the second infiltration neutrophils were 4.7 times more abundant than during the first infiltration (6h, 65±0 neutrophils per 0.2mm^2^; 28 days, 306±3 neutrophils per 0.2mm^2^) we normalized the protease expression to the number of neutrophils. Normalized values showed augmented expression of MPO (p = 0.002), cathepsin G (p = 0.022), proteinase-3 (p = 0.06), and elastase (p = 0.04) during the second infiltration, whereas MMP8 and MMP9 were not different (S4A–S4F Fig). Collectively, these data indicate that increased expression of neutrophil proteases, either because of greater neutrophil infiltration and/or increased protease transcription, is associated with increased tissue pathology.

## Discussion

The goal of this study was to determine the contribution of neutrophils to the development of lesions in American cutaneous leishmaniasis. Studies with various *Leishmania* species and animal models showed that neutrophils can contribute to the control or exacerbation of infection, depending on the resistance or susceptibility of the host [21, 25, 41]. Mice are resistant to infection with parasites of the *Viannia* subgenus [42], so we studied the hamster as a model of experimental ACL because it is more permissive and develops cutaneous lesions that closely resemble chronic human ACL lesions [43, 44]. We found that neutrophils infiltrated the site of cutaneous infection in two waves. In the first wave neutrophils were the most frequent cells infiltrating the infection site with *L*. *panamensis* (within 2–6 hours of infection). The magnitude of the first wave of neutrophils was modest and had no apparent effects on parasite burden or clinical outcome of the early lesion. The second wave of neutrophil influx was much more intense than the first, and was associated with increased lesion size, skin ulceration and necrosis, and parasite burden. Neutrophils in the second wave had reduced oxidative function, nitric oxide production and GM-CSF expression. However, they expressed an array of inflammatory cytokines and neutrophil proteases that probably contribute to tissue pathology of the chronic lesion.

The early neutrophil infiltration (first wave) during the first hours of infection was recognized in mouse models of cutaneous and visceral leishmaniasis infected by sand fly bite or needle inoculation [2, 3]. We found that reduction of this early neutrophil infiltration caused a transient increase in parasite burden of macrophages during the first hours of infection without evident clinical or parasitological effects on the early lesions. A similar result was obtained in a mouse model of visceral infection, which showed that the depletion of neutrophils led to a rapid increase in the early splenic parasite burden but had no effect on the chronic disease [45]. Our failure to observe early parasite control contrasts with studies that demonstrated Leishmania killing capacity of neutrophils [13, 15, 23], as early as 3 hours post-infection [14] However, it agrees with observations indicating that *L*. *donovani* is able to safely survive in the phagosomal compartment during 24-48h [16]. It was demonstrated that *Leishmania* survival could be favored by a lengthened life span of infected neutrophils (from 2–6 hours to 42 hours) or by escaping within apoptotic neutrophils that are phagocytized by macrophages [2, 4]. Even though our results showed that the lesion onset was not affected by early depletion of neutrophils it seems plausible that the extended life span of neutrophils or delayed apoptosis contributed to the accumulation of neutrophils and parasite survival in the chronic lesions.

A second wave of neutrophils occurred after 19 days p.i. A similar second wave of neutrophils was also observed in the infiltrate of ear lesions of C57BL6 mice infected with *L*. *major* [19]. The second wave of neutrophils was significantly correlated with necrosis and ulceration of lesions. This association was supported by the reduction of necrosis and ulcer when neutrophils were depleted (16–45 days p.i.) and an increase in necrosis and ulceration when neutrophil recruitment to the lesion was enhanced. To our knowledge, this is the first study to show the relationship of neutrophil recruitment with ulceration and necrosis in ACL. Although neutrophils were associated with inflammatory cytokines and neutrophil protease expression the mechanisms by which neutrophils promote ulceration and necrosis needs further exploration.

Our results suggest that macrophage but not neutrophil infiltration was associated with lesion onset in this susceptible model of *L*. *Viannia* spp. infection. This notion is supported by: a) infiltration of the skin infection site by macrophages was coincident with lesion onset; b) inhibition of neutrophils at the moment of the infection did not modify the outcome of lesion development; c) uninfected and infected macrophages, but not neutrophils, positively correlated with lesion size. Concurrent with the second wave of neutrophil infiltration, the lesions contained abundant infected macrophages, and extracellular parasites. These findings are in agreement with previous observations of neutrophils associated with parasitized macrophages and an abundance of free amastigotes in the extracellular fluid of human CL lesions [10]. Recruitment of neutrophils to inflamed lesions could be induced either by free amastigotes, acting as chemoattractants [46], or by inflammatory macrophages. Macrophages infected with *L*. *panamensis* isolated from patients with chronic lesions express high levels of CXCL2 and CXCL8 (IL-8), which are chemoattractants for neutrophils [47]. In the present study CXCL2 also was induced by the infection, with similar levels of expression between the first and second waves. This suggests that CXCL2 was not responsible of the enhanced neutrophil recruitment of the second wave. It is possible that chemotactic factors produced by the parasites themselves [45] and inflammatory cytokines [48, 49] could be a stimulus for the massive recruitment of neutrophils in the more heavily parasitized chronic lesions. The second wave of neutrophil infiltration to the lesion, which was coincident with macrophage recruitment, mimics other cutaneous inflammatory diseases where neutrophil products, such as azurocidin, facilitate recruitment of monocytes into the inflamed tissue [50]. Neutrophil proteases such as PR-3 could participate in this process by induction of CXCL-8 and MCP-1 release and ICAM-I expression [51].

Accumulation of neutrophils in chronic lesions could be influenced by delayed apoptosis induced by *Leishmania* infection [4]. Early apoptosis which is considered as an anti-inflammatory process would be opposed to the inflammation observed in CL at the second wave of neutrophils. However, if the capacity of macrophages to clear apoptotic neutrophils is insufficient, dying neutrophils would undergo secondary necrosis, a process known to be inflammatory [52]. Secondary necrosis is characteristic of chronic diseases in which pathology is triggered by massive infiltration of neutrophils that release cytokines and proteases, such as elastase contained in neutrophil granules [53]. We hypothesize that in chronic lesions of CL, macrophages are overwhelmed by the massive number of apoptotic neutrophils. Consequently, the death of neutrophils by secondary necrosis would generate ulceration and necrosis in the CL lesion. The expression of cytokines such as TGFβ, increased in the second wave of neutrophils, would suppress the response of macrophages favoring parasite survival [4]. Further enhancement of neutrophil accumulation could be mediated by chemotactic factors stimulated by inflammatory cytokines IL-1β and TNFα [48], which we found increased with infection. Activation of IL-1β, TNFα and TGFβ by neutrophil MMPs [54] would result in added tissue injury. In addition, accumulated neutrophils could contribute to pathology by impairing T cell immune response, engaging suppressive pathways mediated by depletion of L-arginine and down-regulation of TCRζ [55]. It was demonstrated that phagocytosis of apoptotic neutrophils by *L*. *major*-infected neutrophils suppressed TNFα-induced ROS production and antimicrobial function [56]. This suggests that limiting TNFα-mediated cell death pathways and ROS production would result in prolonged parasite survival [12, 56].

Inflammatory cytokines IL-1β, TNFα and IL-6 could worsen the inflammation and collaborate with the necrosis and ulceration of the lesion during the secondary wave of neutrophils. These cytokines, expressed in both, acute and chronic cutaneous leishmaniasis [57, 58], are typically observed in inflammatory environments with abundant neutrophil recruitment [58, 59]. Studies suggested that excessive expression of inflammatory cytokines rather than resolving the infection results in pathogenesis. Inflammatory IL-1β was associated with the severe form of difuse cutaneous leishmaniasis caused by *L*. *mexicana* [60]. The pathology mediated by CD8 T cells was associated with neutrophils which expressed approximately 70% of IL-1β present in the lesion in a model of CD8 T cell-reconstituted RAG mice infected with *L*. *braziliensis* [61]. The Th17 cytokine IL-17 produced by lymphocytes (and also by neutrophils) was responsible for neutrophil recruitment and lesion development at 6 weeks post-infection of Balb/c mice infected with *L*. *major* [49]. Tissue destruction in mucosal leishmaniasis was also associated with the Th17-regulated cytokines IL-1β, IL-6, TGFβ, IL-23 [59, 62]. Remarkably, neutrophils were surrounding necrotic and perinecrotic areas of Th17-mediated inflammation characterized by abundant neutrophil elastase, myeloperoxidase and MMP-9 [59]. Another inflammatory cytokine, TNFα, which is activated by neutrophil serine proteases is associated with large and ulcerated lesions in CL patients [54, 63]. On the other hand, the activation cascade triggered by these inflammatory cytokines can be also down-modulated by the infection impairing the downstream signaling that kills the parasite. Protein tyrosine phosphatases, e.g. (PTP) SHP-1 expressed in infected phagocytes, dephosphorylate, inhibiting the downstream signal transduction of MAP kinase, JAK/STAT and PI3K pathways, resulting in parasite survival [58, 64]. Together, these studies indicate that excessive inflammatory environment orchestrated by neutrophils, monocytes and T cells harm the tissues resulting in necrosis, ulceration and ineffective parasite killing.

Elimination of the second wave of neutrophil infiltration by cell depletion led to a decrease in intralesional parasite burden, suggesting that these neutrophils directly or indirectly favored parasite survival. The observed decrease in the oxidative capacity of neutrophils at this time point, compared with those of the first wave, suggests the second-wave neutrophils provide a permissive host cell for parasite expansion and promote the establishment of chronic lesions. Although it is well established that proteases released by neutrophils and extracellular traps (NETs) can directly and indirectly kill parasites [15, 65] our results suggest that these mechanisms are ineffective and that neutrophils have a detrimental role, possibly through facilitating the infection of macrophages [16, 18, 20, 21] and impairing dendritic cell function [19, 20, 25]. Since GM-CSF can induce oxidative killing in neutrophils through NADPH oxidase [66] the low lesional GM-CSF may contribute to impaired ROS production by the second wave neutrophils. Further studies are necessary to define the dynamic interaction of neutrophil proteases, activation of inflammatory cytokines and parasite killing in CL.

The massive recruitment of neutrophils with high protease expression is also likely to contribute to tissue damage. In agreement to this observation, neutrophils express MPO, neutrophil elastase and MMP9 at the edge of ulcers and necrotic areas in lesions of patients with mucosal leishmaniasis, indicating their potential involvement in lesion ulceration [59]. Neutrophils were shown to damage the basement membrane underneath the epithelium of the skin, causing keratinocyte death mediated by Fas ligand [67, 68]. Ulcerated lesions are typically associated with a high degree of inflammatory infiltrate which potentially cause tissue damage. Proteases of neutrophils such as proteinase-3 activate pro-inflammatory cytokines (TNFα, IL-1β, and IL-18) [54] that promote inflammation [69, 70] and potentially contribute to ulceration, and necrosis [59, 67, 71]. Histones and MPO released in neutrophil NETs could also mediate activation of IL-1β, resulting in more inflammation and tissue injury [71].

Other studies showed that lesion ulceration and necrosis was associated with recruitment of CD8+ T cells expressing granzyme B [72, 73], which can cause tissue injury. Although we observed that lymphocytes were constant throughout the infection we cannot rule out the possibility that cyclophosphamide or fMLP could have had an indirect effect on the number or phenotype of lymphoid cells (independent of neutrophil modulation). The current lack of validated antibodies specific to hamster T cells limits investigation of the role of T cell subsets in the pathogenesis of CL in this model.

Overall, results from this work indicate that the protective response of neutrophils in ACL is transient and limited to the initial phase of the infection. The second wave of neutrophil infiltration that occurs after the lesion onset has a more prominent role in the pathogenesis of disease, contributing to inflammation, parasite burden, ulceration, and necrosis of the lesion. Therapeutic approaches could consider the inhibition of neutrophils together with the administration of anti-leishmanial drugs to accelerate healing and ameliorate the disfiguring sequelae of ACL.

## Supporting information

S1 Fig Macrophages but not neutrophils were associated with the lesion size in hamsters infected with *L*. *V*. *panamensis*. (A), Abundance of neutrophils plotted against lesion size index (observed lesion size/size before infection); (B), Abundance of macrophages plotted against lesion size index. Number of cells in sections of skin lesions (from 0 to 45 days post-infection) stained with H&E and evaluated in a standard area of 0.21mm^2^at 100X magnification, n = 2 hamsters per point.
(PDF)

S2 Fig Validation of methods to recruit or decrease neutrophils in a hamster dermal air pouch model. (A), Evaluation of methods to deplete neutrophils. Gr-1 monoclonal antibody (clone RB6-8C5, 2.5–5 μg/hamster/1ml ID) and a rabbit polyclonal antiserum against hamster neutrophils (poly, 50–100 μL/hamster/ID) reduce infiltraiton of neutrophils to the infected air-pouch (6 h). Gr-1 antibody reduced neutrophil, but also significantly reduced macrophage infilatration, whereas the anti-hamster neutrophils was specific for the neutrophil population compared with the control serum (Ctr); (B), Evaluation of chemical inhibitors to inhibit recruitment of neutrophils: Aminophylline (Am, 50–800 mg/kg), Metamizole (Met, 500–1,000 mg/kg), and Cyclophosphamide (Cycloph, 200–400 mg/kg) given by the oral route (24h prior infection of the air pouch). Determined using the infected air-pouch model (6 h post-infection); (C), Time course kinetics of depletion of neutrophils with the polyclonal anti-serum and Cyclophosphamide (200 mg/kg); (D), Chemical methods to increase neutrophil recruitment: Lipopolysaccharide (LPS, 250–500 μg/intradermally), N-Formylmethionyl-leucyl-phenylalanine (fMLP, 25 μg/ID) and Di-Nitro-chloro-benzene (DNCB, 0.05–0.1 μg/ID). Evaluated by the dermal air-pouch technique, n = 4–8 hamsters per group: A-C, ***p <0.001, ** p<0.01, Tukey-Kramer Multiple Comparisons Test against corresponding control); D, * p = 0.006, T test, PBS vs. fMLP; (E), Representative histopathology of tissue sections (100X) stained with H&E obtained from hamsters infected in the snout with *L*. *V*. *panamensis* and evaluated 6h p.i. (i) Controls (PBS), (ii) Increased neutrophils (fMLP), (iii) Decreased neutrophils (cyclophosphamide). Black arrows point the presence of neutrophils in reticular dermis and hypodermis.
(PDF)

S3 Fig Response of neutrophils to *in vitro* infection of *Leishmania*. Neutrophils were infected in vitro with CSFE labeled *L*. *panamensis* promastigotes during 20 min. (A), Proportion of infected neutrophils (*p = 0.04, Unpaired T test); apoptotic neutrophils; (B), (*p = 0.01, Unpaired T test) and (C), dead neutrophils (**p = 0.002, Unpaired T test). Determined by flow cytometry.
(PDF)

S4 Fig Protease expression normalized to the number of neutrophils in cutaneous lesions of hamsters infected with *L*. *V*. *panamensis*. (A-D), Expression of myeloperoxidase, cathepsin G, matrix metallopeptidase 8 (MMP8), proteinase-3, neutrophil elastase and matrix metallopeptidase 9 (MMP9) in the infection site of hamsters infected with *L*. *V*. *panamensis*. Evaluated during the first wave of neutrophil infiltration (1^st^ wave, 6h p.i.) or during the second wave of neutrophil infiltration (2^nd^ wave, 28d p.i.). Normalized value = qPCR Fold change with reference to uninfected skin / number of neutrophils. (MPO: p = 0.002; cathepsin G: p = 0.022; proteinase-3: p = 0.06; elastase: p = 0.04, Mann-Whitney Test). n = 5–7 lesions per group.
(PDF)

S1 Table Primers used to detect the expression of hamster cytokines and neutrophil proteases in skin lesions. (PDF)
