# Supplementary figures and images for: A secondary wave of neutrophil infiltration causes necrosis and ulceration in lesions of experimental American cutaneous leishmaniasis
Source: PLoS One. 2017 Jun 7;12(6):e0179084. doi: 10.1371/journal.pone.0179084 (PMC5462435; doi:10.1371/journal.pone.0179084)

**A**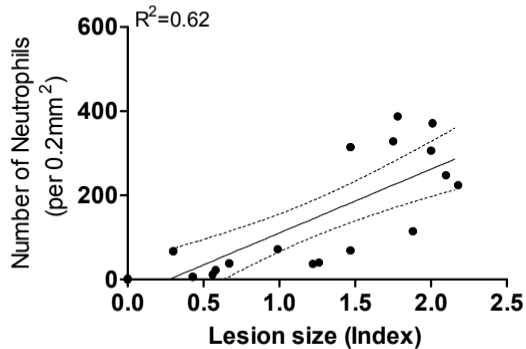**B**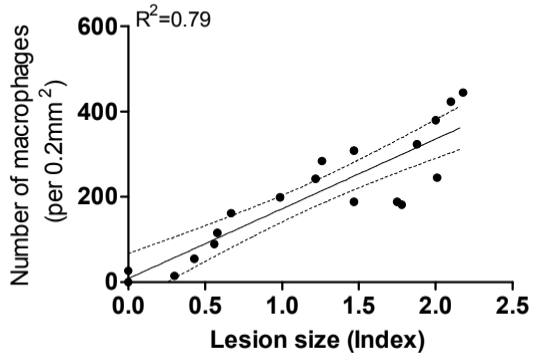

Supplement: S1 Fig — (A), Abundance of neutrophils plotted against lesion size index (observed lesion size/size before infection); (B), Abundance of macrophages plotted against lesion size index. Number of cells in sections of skin lesions (from 0 to 45 days post-infection) stained with H&E and evaluated in a standard area of 0.21mm2at 100X magnification, n = 2 hamsters per point. (PDF) [file pone.0179084.s001.pdf]

**A**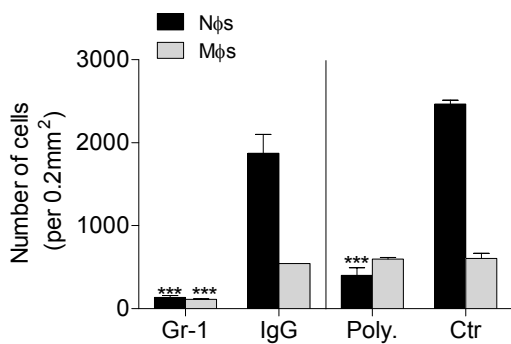**B**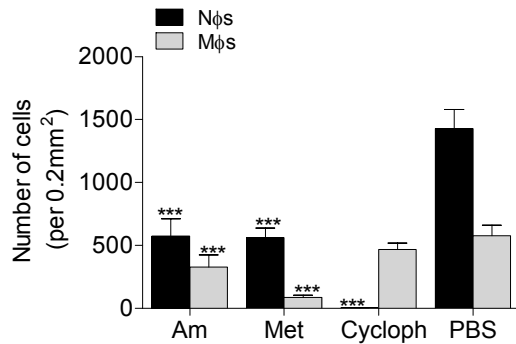**C**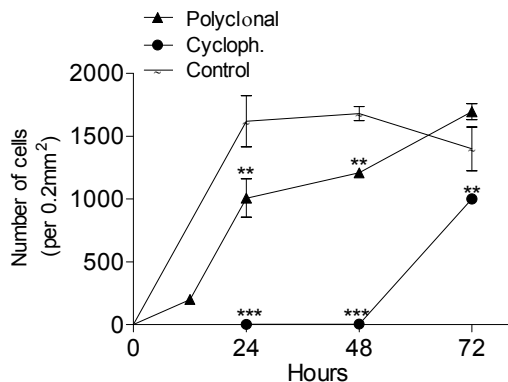**D**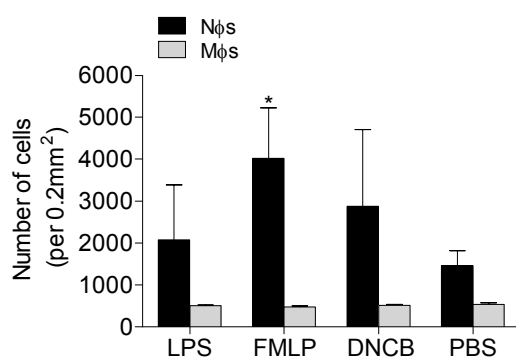**E**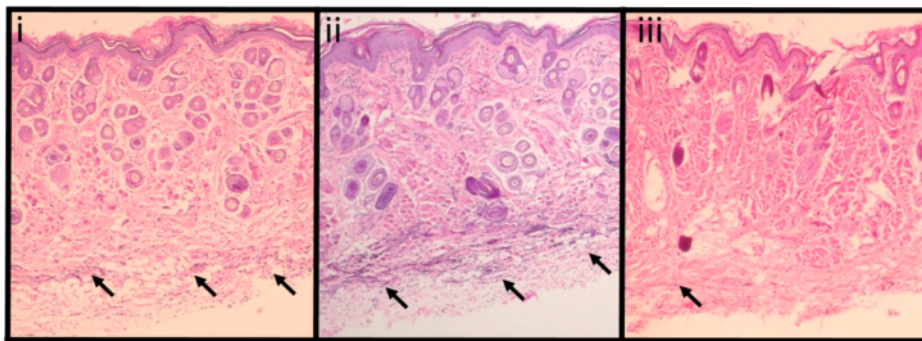

Supplement: S2 Fig — (A), Evaluation of methods to deplete neutrophils. Gr-1 monoclonal antibody (clone RB6-8C5, 2.5–5 μg/hamster/1ml ID) and a rabbit polyclonal antiserum against hamster neutrophils (poly, 50–100 μL/hamster/ID) reduce infiltraiton of neutrophils to the infected air-pouch (6 h). Gr-1 antibody reduced neutrophil, but also significantly reduced macrophage infilatration, whereas the anti-hamster neutrophils was specific for the neutrophil population compared with the control serum (Ctr); (B), Evaluation of chemical inhibitors to inhibit recruitment of neutrophils: Aminophylline (Am, 50–800 mg/kg), Metamizole (Met, 500–1,000 mg/kg), and Cyclophosphamide (Cycloph, 200–400 mg/kg) given by the oral route (24h prior infection of the air pouch). Determined using the infected air-pouch model (6 h post-infection); (C), Time course kinetics of depletion of neutrophils with the polyclonal anti-serum and Cyclophosphamide (200 mg/kg); (D), Chemical methods to increase neutrophil recruitment: Lipopolysaccharide (LPS, 250–500 μg/intradermally), N-Formylmethionyl-leucyl-phenylalanine (fMLP, 25 μg/ID) and Di-Nitro-chloro-benzene (DNCB, 0.05–0.1 μg/ID). Evaluated by the dermal air-pouch technique, n = 4–8 hamsters per group: A-C, ***p <0.001, ** p<0.01, Tukey-Kramer Multiple Comparisons Test against corresponding control); D, * p = 0.006, T test, PBS vs. fMLP; (E), Representative histopathology of tissue sections (100X) stained with H&E obtained from hamsters infected in the snout with L. V. panamensis and evaluated 6h p.i. (i) Controls (PBS), (ii) Increased neutrophils (fMLP), (iii) Decreased neutrophils (cyclophosphamide). Black arrows point the presence of neutrophils in reticular dermis and hypodermis. (PDF) [file pone.0179084.s002.pdf]

**A**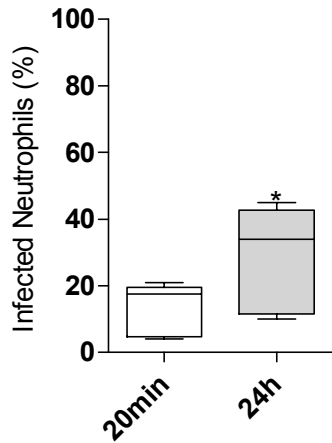**B**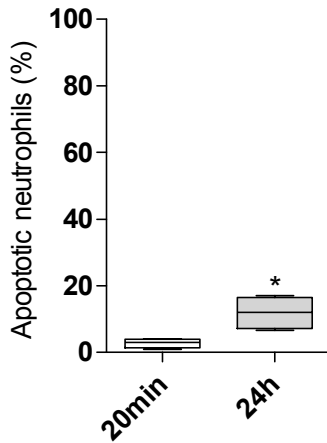**C**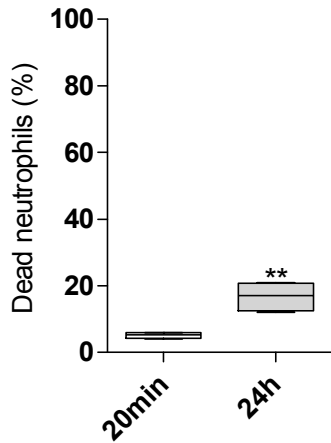

Supplement: S3 Fig — Neutrophils were infected in vitro with CSFE labeled L. panamensis promastigotes during 20 min. (A), Proportion of infected neutrophils (*p = 0.04, Unpaired T test); apoptotic neutrophils; (B), (*p = 0.01, Unpaired T test) and (C), dead neutrophils (**p = 0.002, Unpaired T test). Determined by flow cytometry. (PDF) [file pone.0179084.s003.pdf]

**A**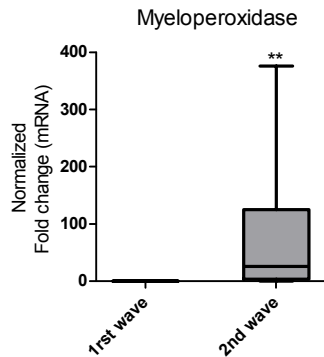**B**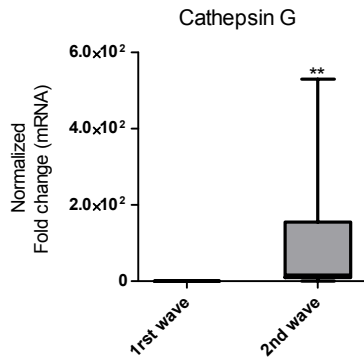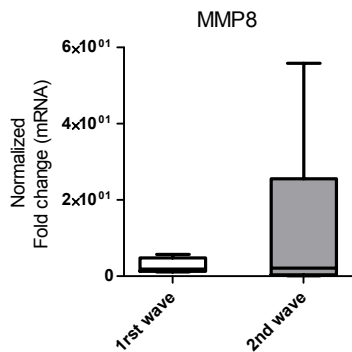**C**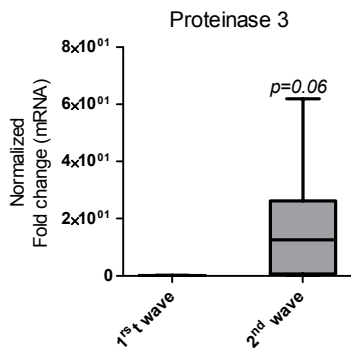**D**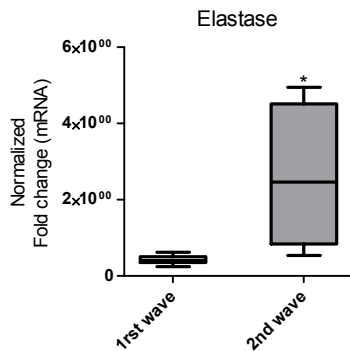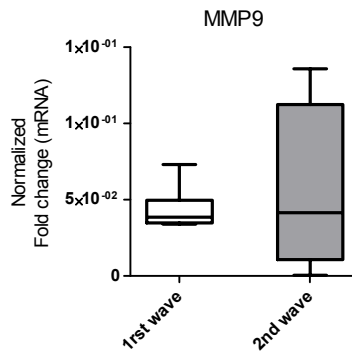

Supplement: S4 Fig — (A-D), Expression of myeloperoxidase, cathepsin G, matrix metallopeptidase 8 (MMP8), proteinase-3, neutrophil elastase and matrix metallopeptidase 9 (MMP9) in the infection site of hamsters infected with L. V. panamensis. Evaluated during the first wave of neutrophil infiltration (1st wave, 6h p.i.) or during the second wave of neutrophil infiltration (2nd wave, 28d p.i.). Normalized value = qPCR Fold change with reference to uninfected skin / number of neutrophils. (MPO: p = 0.002; cathepsin G: p = 0.022; proteinase-3: p = 0.06; elastase: p = 0.04, Mann-Whitney Test). n = 5–7 lesions per group. (PDF) [file pone.0179084.s004.pdf]
