# Supplementary material for: A secondary wave of neutrophil infiltration causes necrosis and ulceration in lesions of experimental American cutaneous leishmaniasis
Source: PLoS One. 2017 Jun 7;12(6):e0179084. doi: 10.1371/journal.pone.0179084 (PMC5462435; doi:10.1371/journal.pone.0179084)
Supplement: S1 Table — (PDF) [file pone.0179084.s005.pdf]

Target gene	Accession Number	Forward	Reverse
Matrix metallopeptidase 9 (MMP-9, Gelatinase B)	XM_005084984	ATGGTGCCCCATGTCACTTT	AGTCTCTCACTGGGGCAGAA
Myeloperoxidase (MPO)	XM_013117645	ACTGGACCCGTTGAGAGATG	GATGCTTTCTCTCCGCTCCT
Collagenase-2 (MMP-8)	AF055671	CCCAATGGAATCCTTGCCCA	TGGGATACATCAAGGCACCG
Cathepsin G-like	XM_005075353	GCCATCCGCCATCCTGATTA	GTTAGTGGCCTCAGGCAGTT
Proteinase-3 (PR-3)	XM_013122983	CACCTTCCTATGTCGGGAGC	AGAATGCCGTCGCAGATCAA
Neutrophil elastase-like	XM_013122977	CCACTGTGTGAACGGCCTAA	GGCTAAGCCATTGAGCTGGA
Colony stimulating factor 2 (Csf2), GM-CSF	XM_005067904.1	AAACCTGAACAACACCCCTGT	GGCCGTTCTCGTACACCTTC
Interleukin 1, beta (IL-1β)	XM_005068610.2	GTCATGAAAGGTGACACGCC	CGCAGACTCAAACTCCACCT

Interleukin 6, (IL-6)	AB028635.1	ACCCTGGCTGTATGGACAATG	AGTCCAGAAGACCAGAGGTGA
C-X-C motif chemokine 2-like (CxCL2)	XM_005068086.2	TTTCAAGACCATCCAGAGCTT	GGGCTTCAGGATCGAGACAAA
Tumor necrosis factor-alpha (TNF-α)	AF046215.1	AGGGAAGAGAAGTTCCCCAAC	TCCACTTGGTGGTTTGCTACA
Transforming growth factor-beta (TGF-β)	XM_013125593.1	AACGACATTCGGGAAGCAGT	ACGCCAGGTATTGTTGCTGT
colony stimulating factor 3 (granulocyte) (Csf3) (G-CSF)	XM_005076035.1	AGCTTCCTGCTGAAGTCCTTGG	GCACAGCTTGTAGGTAGCACAC
Interleukin 18 (IL-18)	XM_013112955.1	GGCTGCCACCTTCTACAACT	GGGCCTTCTTCATGTTTGGC
